# 

**Published:** 1862-01

**Authors:** 


					﻿New Series, Vol. 5, No. 1.
Whole Series, Vol. 19, No. 1.
THE CHICAGO
MEDICAL JOURNAL.
DANIEL BRAINARD, M. D.,
J. ADAMS ALLEN, M. D.,
Editors and Proprietors.
JANUARY, 1862.
CHICAGO :
TlGOTT, BOOK AND JOB PRINTER, 180 CLARK STREET.
1862.
				

